# CNN-Based QR Code Reading of Package for Unmanned Aerial Vehicle

**DOI:** 10.3390/s23104707

**Published:** 2023-05-12

**Authors:** Szu-Yueh Yang, Hsin-Che Jan, Chun-Yu Chen, Ming-Shyan Wang

**Affiliations:** Department of Electrical Engineering, Southern Taiwan University of Science and Technology, Tainan 710, Taiwan; da720201@stust.edu.tw (S.-Y.Y.); 4a92c063@stust.edu.tw (H.-C.J.); 4a92c087@stust.edu.tw (C.-Y.C.)

**Keywords:** unmanned aerial vehicle, convolutional neural network, PID control, Sobel edge computing

## Abstract

This paper plans to establish a warehouse management system based on an unmanned aerial vehicle (UAV) to scan the QR codes printed on packages. This UAV consists of a positive cross quadcopter drone and a variety of sensors and components, such as flight controllers, single-board computers, optical flow sensors, ultrasonic sensors and cameras, etc. The UAV stabilizes itself by proportional-integral-derivative (PID) control and takes pictures of the package as it reaches ahead of the shelf. Through convolutional neural networks (CNNs), the placement angle of the package can be accurately identified. Some optimization functions are applied to compare system performance. When the angle is 90°, that is, the package is placed normally and correctly, the QR code will be read directly. Otherwise, image processing techniques that include Sobel edge computing, minimum circumscribed rectangle, perspective transformation, and image enhancement is required to assist in reading the QR code. The experimental results showed that the proposed algorithm provided good performance of a recognition rate of 94% for the stochastic gradient descent (SGD) and 95% for Adadelta optimization functions. After that, successful QR code reading was presented.

## 1. Introduction

Industry 4.0 was proposed at the Hannover Messe in 2011 [1]. Its main concept is smart manufacturing, which includes artificial intelligence (AI), Internet of Things (IoT), and big data application technology. Driven by the trend of Industry 4.0, smart factories and automatic storage have also become trends, and the latter will also indirectly drive the demand for robots. The design and development of unmanned aerial vehicles (UAVs) or flying robots is growing rapidly. According to a report from Tractica [2], the commercial application of UAVs will push commercial-grade drones from 80,000 in 2015 to more than 2.6 million in 2025, and the annual revenue of commercial drone hardware sales will reach nearly 4 billion U.S. dollars. It is clear that commercial drone services (Drone as a Service, DaaS) will provide considerable business opportunities. The main areas of commercial services include shooting, entertainment, surveying and mapping, aerial operations and other services, and a small part includes survey and disaster relief, early warning systems, data collection and analysis, and environmental monitoring, etc.

The warehouse management system (WMS) is generally divided into warehousing, data creation, packaging, shipment, delivery, and purchase. When operators need to purchase and ship goods, they often need to know the quantity of goods in the warehouse. The traditional warehouse inventory is time consuming and labor intensive. In 2018, Worldwide Chain Stores (WCS) planned to let drones enter the warehouse management market and cooperated with a drone scanning technology company to provide retailers with drone warehouse management solutions [3]. Now, WCS uses the latest drone platform, scanning and communication technologies to integrate WMS and Excel to provide a revolutionary solution for scanning products and pallets in the warehouse, and uses the scanner on the drone to scan the barcode on each pallet, which is 50 times faster than manual. However, the operation needs manual control for UAV.

In addition, in recent years, the rights and interests of workers have been paid more and more attention, but occupational accidents still emerge endlessly every year. The Occupational Safety and Health Administration (OHSA) and the US National Institute for Occupational Safety and Health (NIOSH) published their respective researches on warehousing safety in 2019 [4] and 2013 [5], all of which mentioned that the improper placement and the offset of the center of gravity of goods are one of the main reasons for storage safety accidents.

At present, many studies on UAV storage inventory are carried out with radio frequency identification (RFID) technology, for example: Fernández-Caramés [6], Ong [7], Chin [8], and Huang [9]. However, although RFID can easily identify the content of the barcode, it cannot check whether the package has potential risk factors due to improper placement.

Machine vision is one of the most important parts of artificial intelligence applications. Two-dimensional images can only provide plane information of the object, while three-dimensional images can provide three-dimensional information of the object, but both have the property of rotational invariance. To infer the 3D position and rotational angle of an object from a 2D image, the following methods can be applied: scale-invariant feature transform (SIFT) [10], Hough transform [11], Harris corner detection [12], and so on. In the process of establishing the training set, the concept of plane rotation and three-dimensional rotation is used at the same time. Some training data sets and methods for identifying object rotation are available for experiments in Li [13], Li [14], Diego Marcos [15], Asako [16], and Shi [17]. The rotated MNIST dataset, randomly rotated Imagenet dataset, rotated ShapeNet dataset, and multi-view images of objects are used. In addition, recurrent convolutional neural network (RCNN), multi-view convolutional neural network (MVCNN), and image projection are also for recognition of rotational objects.

At present, artificial intelligence (AI) algorithms have been applied to many fields. There are four classifiers, *k*-nearest neighbor (KNN), support vector machine (SVM), kernel extreme learning machine (KELM), and random forest (RF), used to classify non-severe depression patients and normal subjects by Li et al. [18]. Zhao et al. [19] proposed the elastic net broad learning system and grey wolf optimization algorithms to overcome unsatisfactory prediction and long training time by traditional regression prediction algorithms and achieved a better prediction result of bearing performance. A new bearing fault diagnosis method based on joint distribution adaptive and deep belief network with improved sparrow search algorithm was proposed by Zhao et al. [20] to effectively solve the learning problem of an inconsistent distribution of training data and test data. Moreover, many papers [21,22,23,24,25] have pointed out that convolutional neural networks (CNNs) can solve different application problems in the application of image classification. These papers have discussed that CNNs can achieve the application of image classification and recognition by analyzing images with shape and motion characteristics or directly relying on the self-identification ability of the neural network model. Garbage classification [26], cloud classification [27], clothing classification [28], fruit quality classification [29], and housing classification [30] are the application examples. In addition, a CNN-based hybrid tracking algorithm was developed to robustly track multiple high-speed moving objects simultaneously in [31].

This paper adopts the CNN method and image recognition to classify the placement angles of the packages in the shelf. It cannot only help to identify the QR code, but also can make an assessment and warning of potential risk factors such as improper scanning and placement. This paper will provide some results for advanced study.

- A UAV is designed, which includes a positive cross quadcopter drone and a variety of sensors and components, such as flight controllers, single-board computers, optical flow sensors, ultrasonic sensors and cameras, etc.
- The UAV is successfully stabilized by PID control even when disturbances occurr.
- The placement angle of the package can be accurately classified by CNN. Optimization functions, such as SGD, RMSprop, Adadelta, and Adam, are applied to improve the system performance and show the recognition rates of 94%, 92%, 95%, and 93%, respectively. If the angle is not 90°, a warning will be issued to prompt the management personnel to handle it to avoid accidents or losses. In addition, image processing is required to assist in reading the QR code, including the use of Sobel edge computing, minimum circumscribed rectangle, perspective transformation, and image enhancement. Successful QR code reading is also provided.

In this paper, Section 2 introduces the unmanned aerial vehicle system design. CNN is briefly introduced in Section 3. Experimental results are shown in the next Section 4. Finally, the work and suggestions given for potential future work are concluded in Section 5.

## 2. Unmanned Aerial Vehicle System

**Figure 1:**
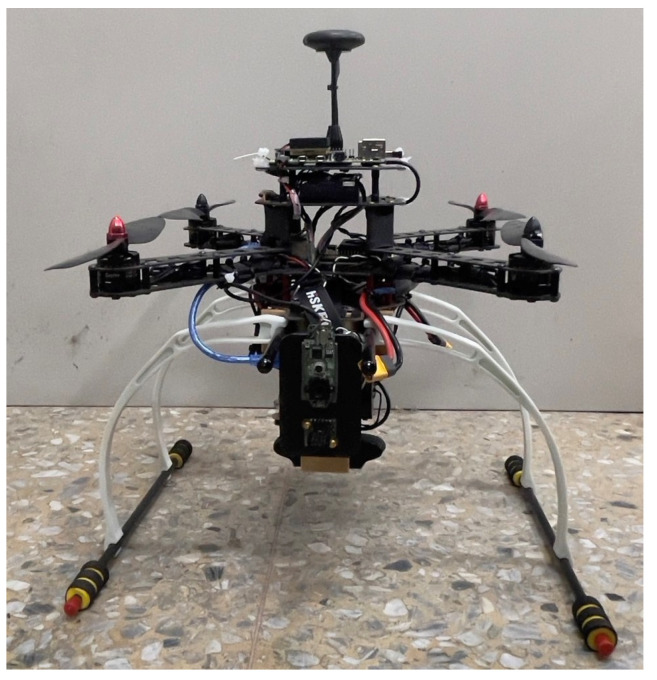
The picture of the unmanned aerial vehicle.

The four-axis quadrature drone is the most commonly used type of UAV. It uses four rotors to generate lift and make the UAV take off and land vertically, hover, and fly. In this paper, a positive fork four-axis UAV with a wheelbase of 38 cm is designed. The flight controller Pixhawk 4 (Holybro Pixhawk 4, https://holybro.com/) and two Raspberry Pi 4 are equipped with an optical flow sensor (Hex Hereflow, https://hexuav.com/), an ultrasonic sensor (MaxBotix XL-MaxSonar MB1260, https://maxbotix.com/), Logitech C920 and C310 cameras, and other components to realize the drone that shoots images before it reaches the shelf autonomously, as shown in Figure 1.

The control system of the UAV is composed of Raspberry Pi 4-1 and the flight controller, and it communicates with the flight controller through the MAVLink (Micro Air Vehicle Link) protocol in the DroneKit drone development library. Attitude estimation is the key parameter to the control system. Throttle control measures the distance to the ground through the optical flow sensor and maintains it at 1.2~1.5 m through the Alt-Hold flight mode. Through the C920 camera on the gimbal capturing the image of the trajectory line, the offsets of the roll angle and the yaw angle are calculated through image processing and then used by proportional–integral–derivative (PID) control to correct the orientation of the drone.

During the flight, the UAV continuously recognizes the trajectory line at the same time. If the trajectory line has not been recognized and the distance between the ultrasonic measurement and the distance between the UAV and the shelf is greater than 70 cm, the UAV will be given a forward pitch angle. If the trajectory line is recognized and the distance between the drone and the shelf is between 60 and 70 cm, the UAV will enter hovering mode. Before the drone arrives on the shelf, Raspberry Pi 4-1 will send a command to Raspberry Pi 4-2 through a Bluetooth interface. After receiving the command, it will turn on the C310 camera to shoot images via file transfer protocol to transfer the photos to the back-end computer.

PID control is widely used in industrial control [32]. Nowadays, many people also apply PID control to UAVs, such as Lin [33], Yang [34], and Zhang [35]. The PID control is expressed as (1),

(1) $$\mathrm{PID}=K_{p}e\left(t\right)+K_{i}\int_{0}^{t}e\left(\tau \right)d\tau +K_{d}\frac{d}{dt}e\left(t\right)$$

where $K_{p},$ $K_{i}$, and $K_{d}$ represent the proportional gain, integral gain, and derivative gain, respectively.

**Figure 2:**
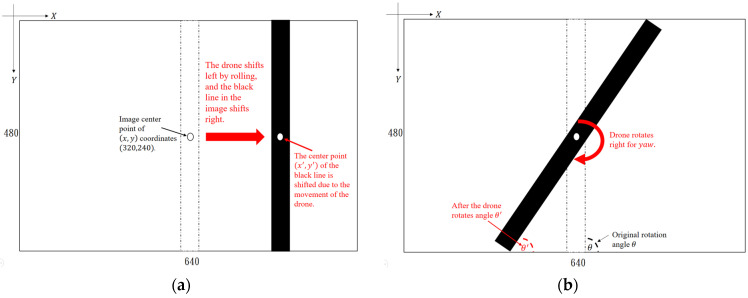
The roll control (**a**) and yaw control (**b**) of the UAV.

In order to keep the drone moving horizontally, it is also necessary to consider issues such as pitch, roll, and yaw angles under different conditions and time. To carry out the roll and yaw control of the UAV by using a shot image with a size of 640 × 480, so that the center coordinates of the image are set to 320 × 280, the minimum bounding rectangle (MBR) is used to obtain the black line image coordinates and calculate the distance between it and the image center coordinates, which is the roll offset distance. The PID control will correct the roll offset of the drone, as shown in Figure 2a. When the black line rotates an angle of θ′, the yaw offset can be known, as shown in Figure 2b. As the distance between the ultrasonic sensor and the object is about 60~70 cm, the UAV will stop executing the pitch command.

## 3. Convolutional Neural Network

A traditional convolutional neural network (CNN) consists of one or more convolutional layers, pooling layers, and fully connected layers (FCs). A convolutional layer adopts an image as its input and is formed by a plurality of different, generally 3 × 3, filters (called convolution kernels) to conduct convoluting operation and then produce different features [36]. The feature map is the output to the next layer through a rectified linear unit (ReLU) for the activation function described as

(2) ReLU(x)=max(0,x)

After down sampling, the outputs are inputted to the fully connected layer for classification [37,38]. The feature map is flattened in the fully connected layer, and the weights are updated in the neural network through backpropagation. The softmax function is used in the output of the fully connected layer and adjust the range of each element in the output vector to locate between 0 and 1, with the sum of all elements being 1. It is described as

(3) $$\sigma {\left(z\right)}_{j}=\frac{e^{z_{j}}}{\sum_{k=1}^{K}e^{z_{k}}},\;j=1,\ldots ,K.$$

The loss function is an important part of the CNN. It is used to measure the inconsistency between the predicted value and the actual label. The robustness of the model increases as the value of the loss function decreases. A cross-entropy algorithm is used to calculate the loss function (4),

(4) $$\mathrm{loss}=-\sum_{i=1}^{N}\sum_{j=1}^{K}t_{ij}\ln\sigma {\left(z\right)}_{ij}$$

where N is the number of samples, K is the number of classifications, and $t_{ij}$ is the actual label. The parameters are updated by

(5) $$z_{\ell +1}=z_{\ell }-\alpha \nabla \mathrm{loss}\left(z_{\ell }\right)$$

where *l* is the number of iterations, ∇loss(ω) is the gradient of the loss function, and α is the learning rate.

The expression for calculating the loss function gradient is as follows:

(6) $$\frac{\partial \mathrm{loss}}{\partial_{z_{i}}}=\frac{\partial \mathrm{loss}}{\partial \sigma {\left(z\right)}_{j}}\frac{\partial \sigma {\left(z\right)}_{j}}{\partial z_{i}}=-\frac{t_{i}}{\sigma {\left(z\right)}_{i}}\times \sigma {\left(z\right)}_{i}\left(1-\sigma {\left(z\right)}_{i}\right)=-t_{i}+t_{i}\sigma {\left(z\right)}_{i}$$

where j means all outputs and i is one of them.

The regularization strategy is to limit the ability of the model through penalty, which adds a norm constraint to the loss function [39]. L1 regularization refers to the sum of the absolute values of each element in the weight vector *w*. Its expression is as follows,

(7) $$L=L\left(\omega \right)+\lambda \sum_{1}^{n}\left|\omega_{i}\right|$$

(8) $$\omega_{i}=\omega_{i}-\eta \left(\frac{\partial L\left(\omega \right)}{\partial \omega_{i}}+\lambda \cdot sign\left(\omega_{i}\right)\right)$$

where L is a function with absolute value sign, λ is the regularization coefficient and η is the learning rate. L2 regularization is the squared root of the sum of squares of the various parameters of the model. Its expression is as follows,

(9) $$L=L\left(\omega \right)+\lambda \sum_{1}^{n}\omega_{i}^{2}$$

(10) $$\omega_{i}=\omega_{i}-\eta \left(\frac{\partial L\left(\omega \right)}{\partial \omega_{i}}+2\lambda \cdot \omega_{i}\right)=\left(1-2\eta \lambda \right)\omega_{i}-\eta \frac{\partial L\left(\omega \right)}{\partial \omega_{i}}$$

Compared with the L2 constraint, the L1 constraint can produce a sparser model. Under a smaller w, it will be reduced to 0, thereby achieving the function of feature selection. That is, L1 has sparsity and can be used for feature selection, while L2 can improve computational efficiency and has an analytical expression, so both can prevent model overfitting.

## 4. Experimental Results

**Figure 3:**
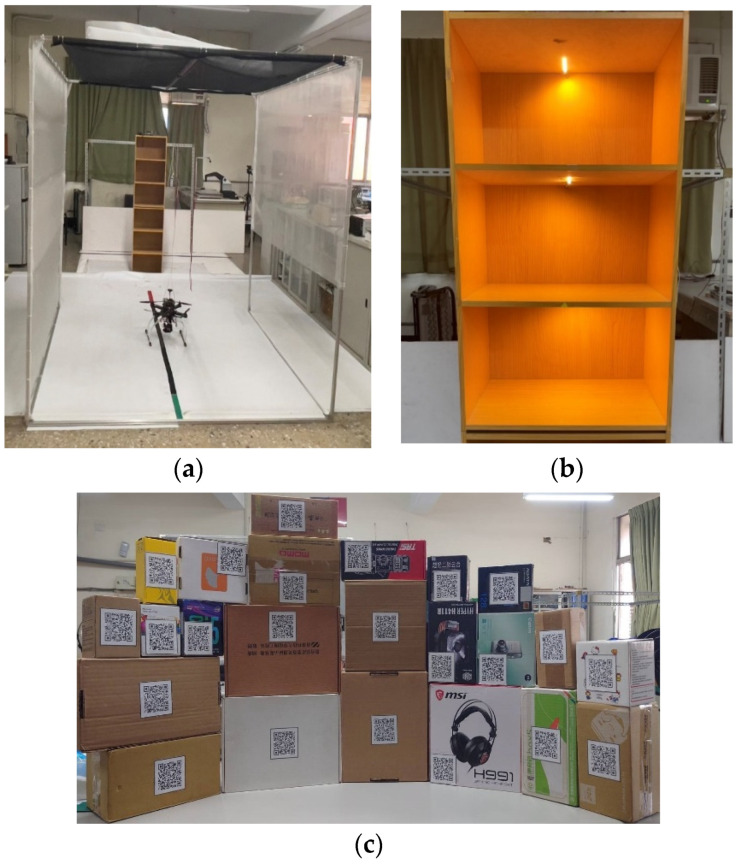
Experimental environment and package placement angle. (**a**) UAV flight field and frame, (**b**) fill light in the cabinet, and (**c**) package type.

In order to explore how to use UAVs to take pictures of packages on the shelves, read the QR code information on the packages, and realize the function of warehouse management, the experimental environment was set up by laying a white canvas with a length of 300 cm and a width of 350 cm on the indoor floor and pasting a black line with a length of 300 cm and a width of 4.5 cm and a red line with a length of 30 cm in the middle of the canvas as the end point, as shown in Figure 3a. In order to ensure the safety of indoor flight, a flight frame was built, and the top and left and right sides of the frame were covered with black gauze and white gauze. It consisted of two identical three-layer cabinets, 160 cm high, 40 cm wide, and 30 cm deep. A yellow light strip was installed in the cabinet to provide supplementary light in the cabinet, as shown in Figure 3b. Different packages are shown in Figure 3c. The size of the QR code on the package was 7 cm in length and width, an outer frame for identification was added on the outside, and the information in the QR code was the website of Southern Taiwan University of Science and Technology and the website of a random shopping website.

**Figure 4:**
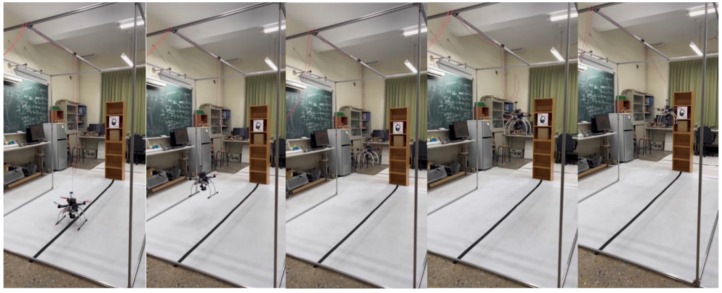
UAV flight process.

Figure 4 shows the five segmented screens of the video of the experiment process during the actual flight. Firstly, the drone was placed on the black line on the ground and prepares to take off, then the drone started to take off and increase its altitude. The optical sensor mounted on the UAV was continuously detecting the height with the ground until the height of the drone was raised to 1.2 to 1.5 m. In addition, the UAV kept moving forward during these processes and detected the distance from the shelf by the ultrasonic sensor. Finally, the flight controller stopped advancing command and kept hovering.

**Figure 5:**
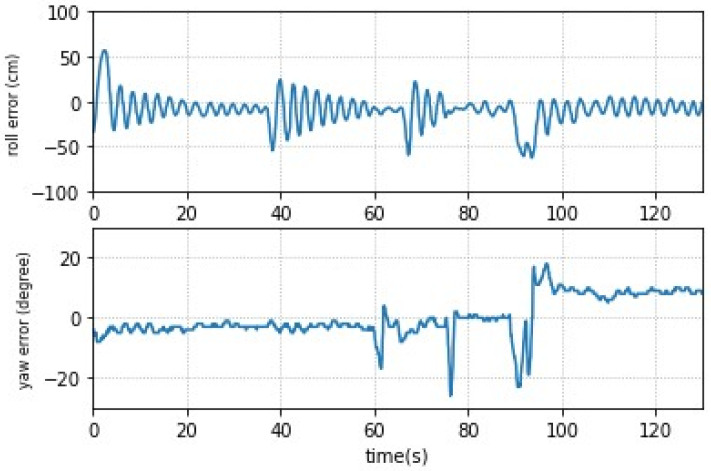
UAV roll and yaw control by PID control.

The experimental results of the UAV flight by PID control are shown in Figure 5. The UAV was placed randomly on the black line on the ground in the beginning, so there would be errors initially. The UAV successfully corrected back to the predetermined trajectory within 20 s after takeoff. In order to test the roll correction ability, the external force disturbance was applied to the UAV during the flight at the instances of 40, 70, and 90 s. There was a deviation due to disturbance, but it was successfully corrected later. Similarly, the external force for yaw control was applied to the UAV during the flight at the instances of 60, 75, and 90 s.

**Figure 6:**
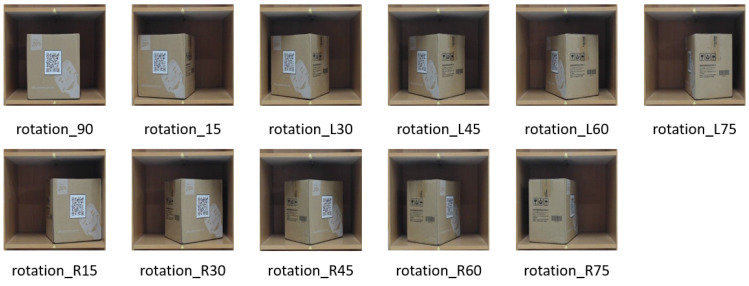
Various rotated packages.

In order to establish the image of the placement angle of the package, the shelf mezzanine was used as the baseline, the middle of the baseline as the origin, and a protractor was used to assist in drawing 90°, left 15, left 30, left 45, left 60, and left 75. The same was true for the right side of the base line. When creating training and test data for the package placement angle, the package needs to be placed based on the base line and the origin, as shown in Figure 6.

**Figure 7:**
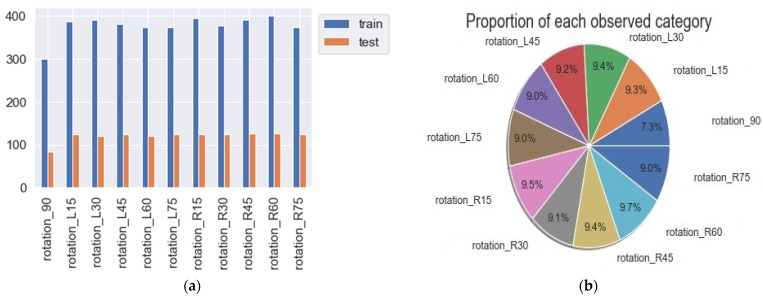
Data volume and distribution. (**a**) Training and testing data volume; (**b**) data volume distribution.

Next, a method that can identify the placement angle of the package is needed. CNN generally uses multiple convolutional layers to extract object features, but the angle of the package has no identifiable features in the image. Therefore, the difference in surface size and outline when the package is placed at different angles may define the package placement angle. Through different shooting heights, distances, offsets, rotations, lighting, contrast, and cropping, more training data can be generated for CNN. In order to make the similarity between the training image and the test image not too high, random deletion is considered, and then 20% of the images are selected as the validation set, and the rest are used as the training set. Figure 7a shows the number of training and validation data for training CNN, and Figure 7b shows the data ratio of images from each angle.

**Figure 8:**
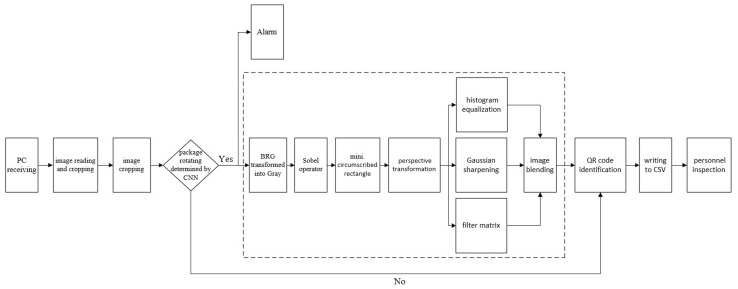
Identification process of a QR code.

After creating a package image, the QR recognition will then be processed, as shown in Figure 8. After the back-end computer receives the image, it will crop the environment image directly and then predict whether the package is rotated through CNN. If there is no rotation, the pyzbar decoder is used to read the package information directly from the image. Otherwise, the QR code image will be corrected back to the forward image through perspective transformation. Then, histogram equalization and other technologies are used to strengthen the outline of the QR code profile, so that the pyzbar decoder can read the package message from it.

**Figure 9:**
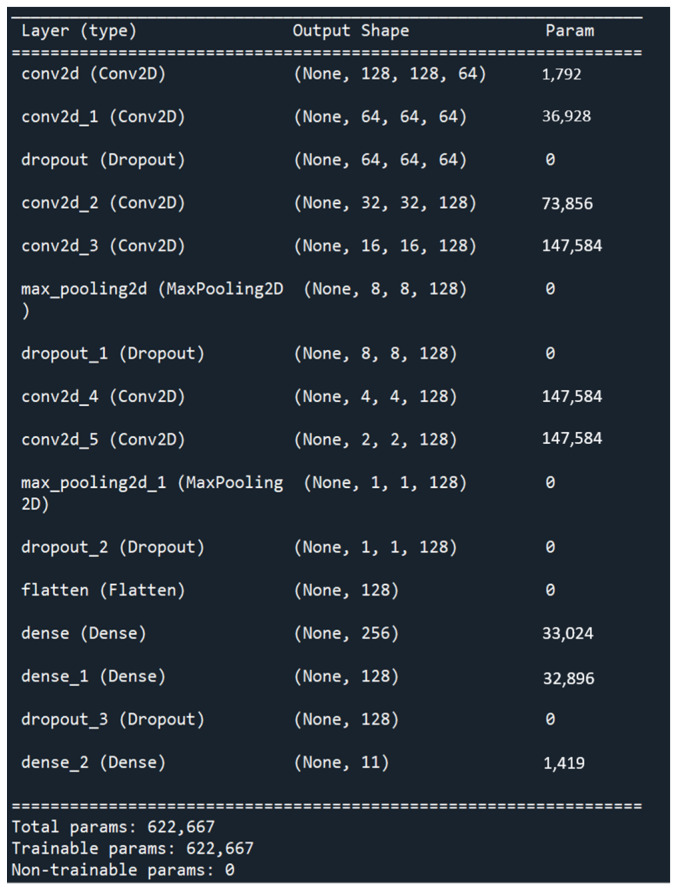
CNN architecture used in this paper.

The computer specifications used in the experiment are: Intel Core i9-9900K 3.6 GHz, GeForce RTX 3080 10G, CUDA (Compute Unified Device Architecture), and the number of training data is 8704. The CNN architecture used in this paper, as shown in Figure 9, contains 6 convolutional layers, 2 pooling layers, 4 Dropout layers, 2 fully connected layers, and 1 SoftMax layer. The Dropout layer is a regularization method that can effectively prevent over-fitting effects. It randomly discards the hidden layer neurons with the probability we set (25%), so that the model does not rely too much on specific neurons, so as to increase the generalization ability of the model, thereby achieving the purpose of preventing overfitting. We add a layer of Dropout before the SoftMax layer because Dropout uses the Bernoulli function to randomly give 0 and 1, so we use 10% Dropout to verify the robustness of the system and then classify 11 kinds of package placement angles through the SoftMax layer. In order to optimize the results of the model, hyperparameters are adjusted and optimized for better performance.

Hyperparameters refer to parameters that are not learned by the model itself, such as the number of hidden layers of the model, optimizer, number of discarded layers, learning rate, regularization coefficient, batch size, training cycle, etc. These parameters can affect the performance and behavior of the model and can be divided into grid search and random search. The grid search will adjust the learning rate, batch size, and regularization coefficient of the model, and different optimization functions are applied for comparison, such as SGD, RMSprop, Adadelta, Adam, Adamax, and Adagrad. Then, the adjusted model is subjected to 10-fold cross-validation to confirm performance and accuracy. K-fold cross-validation divides the original data set into K non-overlapping subsets, and then each subset takes turns as the test set, and the remaining K-1 subsets are used as the training set. The model is trained K times and tested K times, and finally, the average value of the K test results are obtained as an estimate of the model performance. The results are shown in Table 1. Regularization coefficients L1 and L2 of 0.001 reduce the problem of overfitting. The accuracy of various optimization functions in the table is about 90%, and only Adamax has the probability of the accuracy rate less than 10%. For Adagrad, its convergence speed is too slow, and the accuracy rate is lower than other methods. So, these two methods can be excluded.

**Figure 10:**
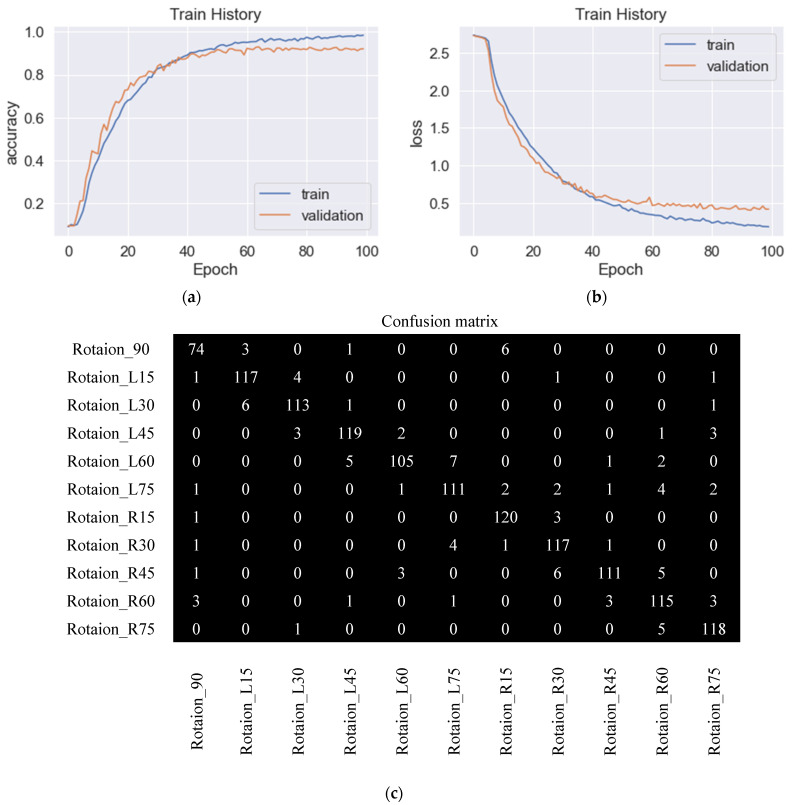
Training and validation of the proposed CNN, performed using SDG. (**a**) Training and validation accuracy with 92.0% accuracy, (**b**) training and validation loss, and (**c**) confusion matrix.

The model trained by SDG had an accuracy rate of 92.0% in identification, and the parameters were set as learning rate 0.01, learning rate decay rate 0.000001 after each parameter update, movement amount 0.9, and Nesterov momentum included. The SDG training process is shown in Figure 10a. The figure describes the accuracy rate during the training and verification process. Figure 10b shows the loss during SDG training, and the accuracy rate did not change much at Epoch 70. Figure 10c is the confusion matrix in which we can find that each angle had mutual confusion.

Table 2 displays the test results of the trained model with 20 test photos for various angles. It was found that three photos were wrongly identified in the recognition of L45° and L60°. The time required to identify a single image of the package excluded the image pre-processing time was about 0.023 s. So, the recognition of the trained model with 20 test photos for one displacement angle needs 0.478 s, about 20 times of 0.023 s, in Table 2. We used time.time() in the python time module to calculate time.

**Figure 11:**
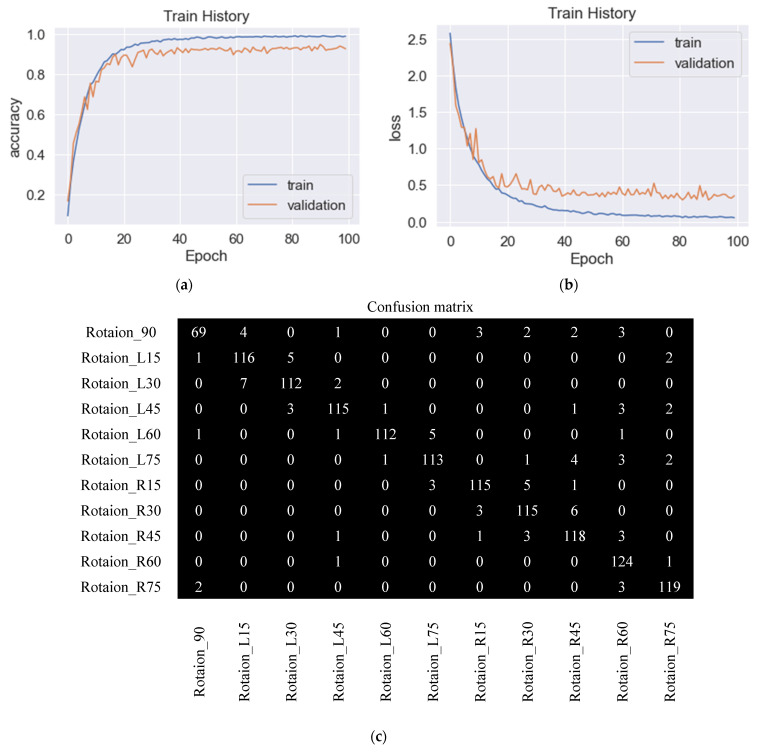
Training and validation of the proposed CNN, performed using RMSprop. (**a**) Training and validation accuracy with 92.9% accuracy, (**b**) training and validation loss, and (**c**) confusion matrix.

**Figure 12:**
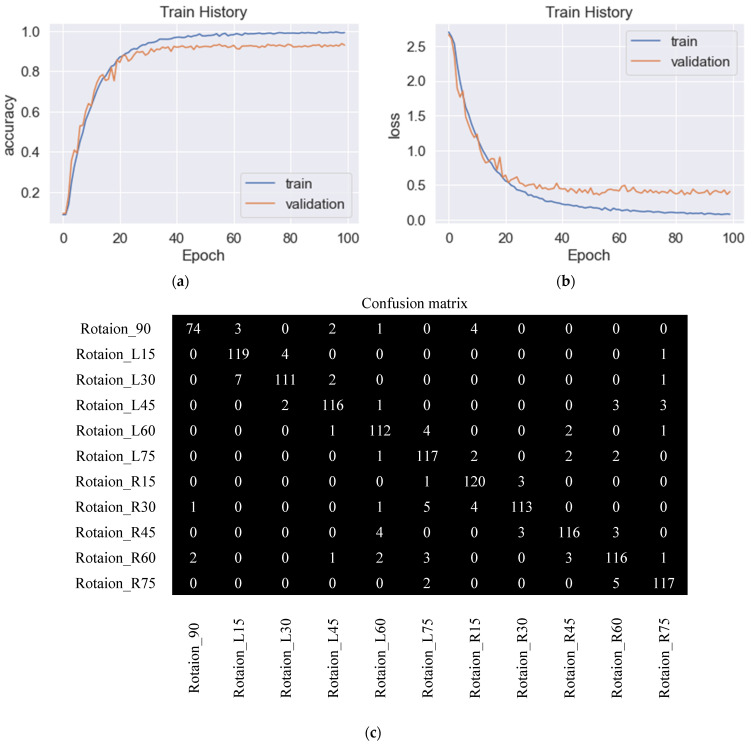
Training and validation of the proposed CNN, performed using Adadelta. (**a**) Training and validation accuracy with 92.9% accuracy, (**b**) training and validation loss, and (**c**) confusion matrix.

**Figure 13:**
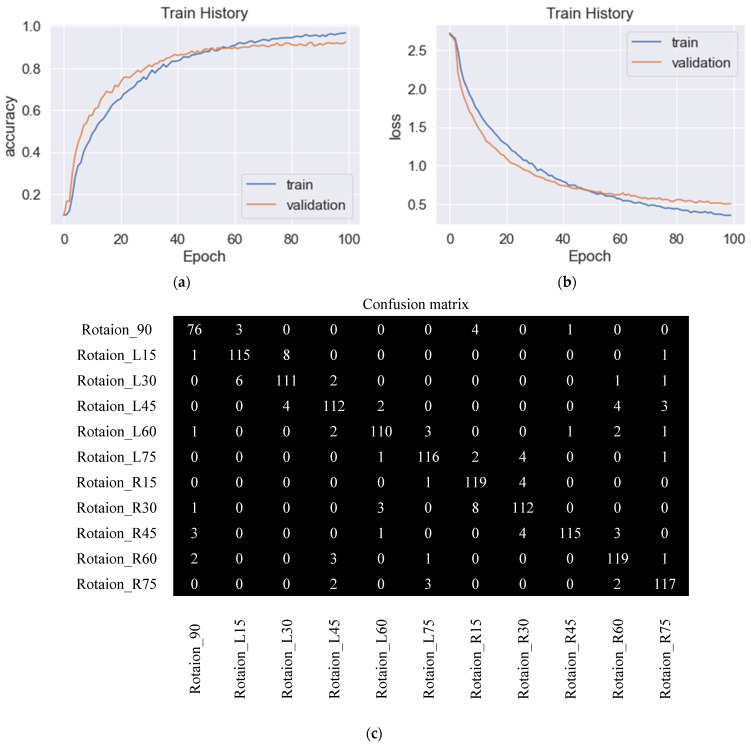
Training and validation of the proposed CNN, performed using Adam. (**a**) Training and validation accuracy with 92.3% accuracy, (**b**) training and validation loss, and (**c**) confusion matrix.

For various optimization functions, their results of accuracy and loss are compared. With the parameters set as learning rate 0.001, the moving average of gradient squared decay rate 0.9, and positive learning rate decay value after each parameter update, Figure 11a shows an identification accuracy rate of 92.8% by RMSprop. Figure 11b shows the loss in process by RMSprop, and there were still slight shocks at the 70th and 90th training stages. Figure 11c shows the confusion matrix. There were situations where various angles were confused with each other, especially at 90° as the most obvious, and a total of 15 images were misidentified for L15°, L45°, R15°, R30°, R45°, and R60°. Figure 12a shows the training process of Adadelta, with an identification accuracy rate of 92.9%. The parameters were set to learning rate 1.0, the moving average of the gradient squared decay rate 0.95, and positive learning rate decay after each parameter update. Figure 12b shows the loss values, and the accuracy rate did not change much from the 50th training stage. The confusion matrix is shown in Figure 12c; there were mutual confusions in various angles, especially at R60° most obviously, and 12 images were misrecognized, which were 90°, L45°, L60°, L75°, R45°, and R75°, respectively. The accuracy rate of 92.3% by Adam is shown in Figure 13a. From the 100th training stage, the accuracy rate did not change much. The parameters were set as the learning rate 0.001, the exponential decay rates of the first-order momentum and the second-order momentum β1, β2 0.9 and 0.999, respectively, and the positive learning rate decay value after each parameter update was also required. The loss process is shown in Figure 13b. The confusion matrix is shown in Figure 13c, and there were various angles in the confusion matrix that still confused each other, especially at L45°. A total of 13 images were wrongly identified as L30°, L60°, R60°, and R75°. It was found that Adam has the slowest convergent rate.

Next, 20 test photos for each performance of the model trained by the above-mentioned various optimization functions is shown in Table 3. The recognition on L60° had a higher error rate, followed by L15°.

From the above, CNN can be used to identify the placement angle of the package. Here, we can apply the angle identification by CNN in two ways:

First: When CNN recognizes that the package is rotated (that is, the placement angle is not 90°), it means that the package may not be placed properly. At this time, the system sends out a warning message to warn the management personnel to arrange it to prevent the package falling from the shelf and causing accidental or unnecessary damage.

**Figure 14:**
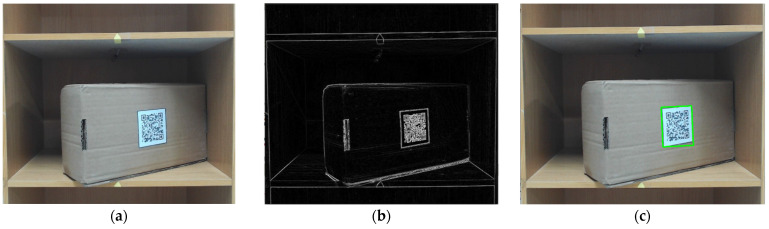

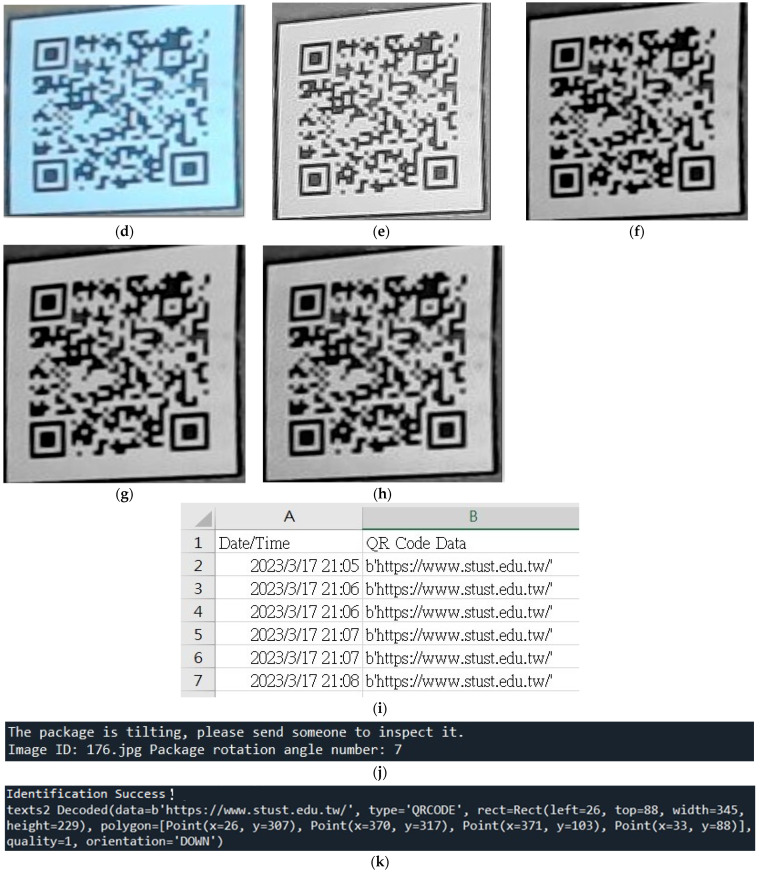
QR code image correction and reading. (**a**) Original image, (**b**) Sobel operator, (**c**) QR code framed by the minimum circumscribed rectangle, (**d**) perspective transformation, (**e**) mask matrix, (**f**) Gaussian blur (sharpening), (**g**) histogram equalization, (**h**) image overlay, (**i**) write the read information to CSV, (**j**) predict that the package is not 90° with a rotation and list a warning, and (**k**) success message.

Second: Since the QR code is printed on the interested surface of the package, it can be inferred that when the package is rotated, that is, the QR code image must also produce a rotation of the same angle. In order to improve the efficiency of the overall work and reduce unnecessary collisions that may be caused by drones looking for the best shooting position of the QR code in the region of interest, it can be used as a decision whether to process the image again. If the CNN recognizes that it is 90°, it means that the package and the QR code image are not rotated, and the UAV can directly read the QR code at this time. Otherwise, the image will be processed to assist in reading the QR code (Figure 14a). The processing process is shown as follows. Firstly, the Sobel edge operation is performed on the original image (Figure 14b) to make the QR code image obvious, and then the QR code image is framed by the minimum circumscribed rectangle (Figure 14c). After that, the skewed QR code image is pulled back to the forward image by perspective transformation for easy reading (Figure 14d). In order to improve the recognition rate, the pictures after the perspective transformation are subjected to various image enhancements, such as filter matrix (Figure 14e), Gaussian sharpening (Figure 14f), histogram equalization (Figure 14g), and image blending (Figure 14h), as shown in Figure 14. Figure 14i shows writing the read information to CSV; predicting the package is not 90° with a rotation and listing a warning is shown in Figure 14j; and Figure 14k shows the success message of QR code reading.

## 5. Conclusions

This study intends to develop a warehouse management system based on UAVs to scan the QR codes printed on the packages. The UAV includes a positive cross quadcopter drone with a wheelbase of 38 cm and a variety of sensors and components, such as flight controllers, single-board computers, optical flow sensors, ultrasonic sensors and cameras, etc. The UAV is stabilized by PID control and takes pictures of the package as it reaches ahead of the shelf autonomously. Through convolutional neural networks (CNN), the placement angle of the package can be accurately identified. Optimization functions, such as SGD, RMSprop, Adadelta, and Adam, are applied to improve the system performance. In terms of the results, two cases should be considered. If the angle is 90°, the QR code can be read directly. Otherwise, image processing is required to assist in reading the QR code, including the use of Sobel edge computing, minimum circumscribed rectangle, perspective transformation, image enhancement, and other steps. Furthermore, the system will issue a warning to prompt the management personnel to arrange the package to avoid accidents or losses and use the placement angle for the QR code map to improve the drone’s work efficiency and reduce unnecessary collisions between the drone and package.

The UAV system can also be applied in monitoring the safety of the warehouse interior and conducting inventory counts. In addition, through the combination of the UAV system and IoT technology, real-time monitoring and management of the entire logistics process can be realized, thereby improving the operational efficiency and quality of logistics.

## Figures and Tables

**Table 1:** The accuracy of various optimization functions confirmed by 10-fold cross-validation.

10-Fold	Optimization Function			
	SGD	RMSprop	Adadelta	Adam
1	89.4%	91.4%	91.9%	90.9%
2	91.0%	93.3%	91.5%	90.9%
3	90.5%	93.0%	91.9%	91.8%
4	90.5%	91.5%	91.5%	90.8%
5	90.4%	92.7%	93.1%	91.9%
6	91.0%	89.7%	92.1%	91.3%
7	90.5%	91.0%	91.7%	90.3%
8	89.4%	90.9%	90.5%	91.5%
9	90.8%	92.4%	91.8%	91.4%
10	90.9%	92.6%	91.8%	90.6%

**Table 2:** Rotation recognition results of 20 packages tested using the SGD method.

No.	Angle of Package (SGD)										
	90°	L15°	L30°	L45°	L60°	L75°	R15°	R30°	R45°	R60°	R75°
1	0	0	0	0	0	0	0	R45°	0	0	0
2	0	0	0	0	0	0	0	0	0	0	0
3	0	0	0	0	0	0	0	0	0	0	0
4	0	0	0	0	0	0	0	0	0	0	0
5	0	0	0	0	0	0	0	0	0	0	0
6	0	L30°	0	0	0	0	0	0	0	0	0
7	0	0	0	0	0	0	0	0	0	0	0
8	0	0	0	0	0	0	0	0	0	0	0
9	0	0	0	L60°	0	0	0	0	0	0	0
10	0	0	0	0	0	0	0	0	0	0	0
11	0	0	0	L30°	0	0	R30°	0	0	0	0
12	L15°	0	0	0	L45°	0	0	0	0	0	0
13	0	0	0	0	0	0	0	0	R60°	0	0
14	0	0	0	0	0	0	0	0	0	0	0
15	0	0	0	0	0	0	0	0	0	0	L75°
16	0	0	0	0	0	0	0	0	0	0	0
17	0	0	0	0	0	0	0	0	0	0	0
18	0	R75°	0	L30°	0	0	0	0	0	0	0
19	0	0	0	0	R60°	0	0	0	0	0	0
20	0	0	0	0	L45°	0	0	0	0	0	0

**Table 3:** Recognition ability of different optimization functions from various angles.

Angles	Optimization Function			
	SGD	RMSprop	Adadelta	Adam
90°	95%	95%	90%	95%
L15°	90%	85%	95%	90%
L30°	100%	95%	100%	95%
L45°	85%	95%	95%	90%
L60°	85%	85%	90%	80%
L75°	100%	95%	100%	100%
R15°	95%	90%	95%	95%
R30°	95%	95%	100%	95%
R45°	95%	90%	95%	95%
R60°	100%	100%	95%	95%
R75°	95%	90%	95%	90%
Average	94%	92%	95%	93%

## Data Availability

Not applicable.
